# MELAS syndrome with rare manifestations misdiagnosed as vasculitis in the absence of lactic acidosis: A case report

**DOI:** 10.1016/j.amsu.2022.104483

**Published:** 2022-08-27

**Authors:** Mohammad Alsultan, Deema Alshaar, Badie Alkhouli, Qussai Hassan

**Affiliations:** a Department of Nephrology, Al Assad and Al Mouwasat University Hospital, Damascus University, Faculty of Medicine, Damascus, Syria; b Department of Histopathology, Al Assad University Hospital, Damascus University, Faculty of Medicine, Damascus, Syria; c Department of Nephrology, Al Assad University Hospital, Damascus University, Faculty of Medicine, Damascus, Syria

**Keywords:** MELAS syndrome, Vasculitis, Uveitis, Lactic acidosis, Parkinsonism, FSGS

## Abstract

**Introduction:**

The syndrome of mitochondrial encephalomyopathy with lactic acidosis and stroke-like episodes (MELAS) is one of the most common inherited mitochondrial disorders.

**Presentation of case:**

A 33- year-old male was admitted due to edema, urinary retention, and reduce urinary output. The medical history included a pigmentary retinopathy (PR) at age of 22 and uveitis at age of 30, which were both treated with prednisolone. At age of 32, unapparent bilateral sensorineural hearing loss (SNHL) and symmetric basal ganglia calcifications were observed in neurologic study, and received prednisolone for the diagnosis of migraine and undefined vasculitis. Also, he described a right transient ischemic stroke (TIA) in the past 4 months. His family history included a dead brother, who had nearly similar components. Physical exam on admission corresponded with parkinsonism. The status points to MELAS but the genetic test was not available. Additional tests were applied, excluding all other disorders. Lactate was normal in serum and CSF. Kidney tests revealed a nephrotic syndrome and glomerulopathy, and the biopsy showed a single hyalinized glomerulus, which most likely suggests focal segmental glomerulosclerosis (FSGS). Muscle biopsy showed ragged red fibers.

**Conclusion:**

Here, we report a challenging case of MELAS syndrome with rare manifestations including uveitis, PR, parkinsonism, and FSGS in the absence of lactic acidosis with unapparent muscle or hearing impairments. Since, clinicians might misdiagnose MELAS as vasculitis or other disorders due to its heterogeneous presentations, a proper investigations should guide the diagnosis of these conditions to reduce the delay of diagnosis and ineffective treatments.

## Highlights

• Clinicians might misdiagnose MELAS as vasculitis or other disorders due to its heterogeneous presentations.
• Muscle biopsy showed ragged red fibers with unapparent muscle impairment.
• MELAS can demonstrate a typical Parkinson's disease with extrapyramidal features.
• Relevant investigations should guide the diagnosis to reduce the delay of diagnosis and ineffective treatments.

## 1 Introduction

Mitochondrial diseases can manifest with a wide range of clinical phenotypes, which presents a significant diagnostic challenge for clinicians [1]. The syndrome of mitochondrial encephalomyopathy with lactic acidosis and stroke-like episodes (MELAS Syndrome) is one of the most common inherited mitochondrial disorders. Approximately, 80% of cases are associated with m.3243A > G mutation, and 10% are related to the m.3271T > C transfer RNA mutation [2]. The typical age of presentation is childhood with 65–76% of affected individuals presenting at or before the age of 20 years and 1–6% after the age of 40 years [2]. The clinical phenotype associated with this syndrome usually includes stroke-like episodes, seizures, migraine-like headaches, sensorineural hearing loss (SNHL), and muscle weakness [1]. Diabetes, peripheral neuropathy, short stature, and psychiatric disorders have also been described [2].

The underlying mechanism of MELAS syndrome remains unknown. Patients with the MELAS found to have marked deficiency in the activity of complex I of the respiratory chain [3]. Tissues with high energy requirements (ie, brain, skeletal muscle) are mostly affected [1]. Therefore previous studies have suggested severe depletion of ATP production due to mitochondrial dysfunction and consequently dysfunction of the Na+/K + -ATPase and Ca2+-ATPase, which impairs plasma membrane potential [4,5]. Other implicating mechanisms are microangiopathy and nitric oxide (NO) deficiency [2].

Here, we describe an unusual case of MELAS syndrome with confusing presentations and multiple organs involvement that has been misdiagnosed as vasculitis in the absence of lactic acidosis. This case report examines one such presentation in line with the SCARE guidelines [6].

## 2 Presentation of case

A 33- year-old male was admitted to our Nephrology Department complaining of edema for a year, urinary retention, and reduce urinary output for the past three months. The past medical history included various hospital admissions and medical records. The patient was diagnosed with pigmentary retinopathy (PR) at age of 22 and uveitis at age of 30, in these occasions, he was treated with high-dose prednisolone with subsequently tapering.

In the past year at age of 32, he had neurology admission for studying headache and vertigo. Neurologic examination was normal except for hypertonia in the upper left limb. MRI showed bilateral basal ganglia calcifications and lacunar infarcts. Also, an unapparent bilateral sensorineural hearing loss (SNHL) was observed. At this point, the patient was diagnosed with migraine and undefined vasculitis and discharged on high-dose prednisolone with subsequently tapering. In the past four months, he described regression of right hemiplegia with dysarthria, which corresponding with TIA, and was put on aspirin, rivaroxaban, and atorvastatin.

His family history included a brother, who had nearly similar components consisting of migraine, uveitis, basal ganglia calcifications, psychotic disorder, and diabetes. Also, the brother had hematologic admission for studying lymphadenopathy and dead due to bloodstream infection at age of 29.

Physical exam on admission was as follows: 158 cm, blood pressure 110\70 mmHg, hyperreflexia and hypertonia in all limbs, cogwheel rigidity, positive Hoffman sign, bradykinesia, mild ptosis, and decrease visual acuity without apparent hearing impairment.

### 2.1 Solving the diagnostic challenge?

**Fig. 1:**
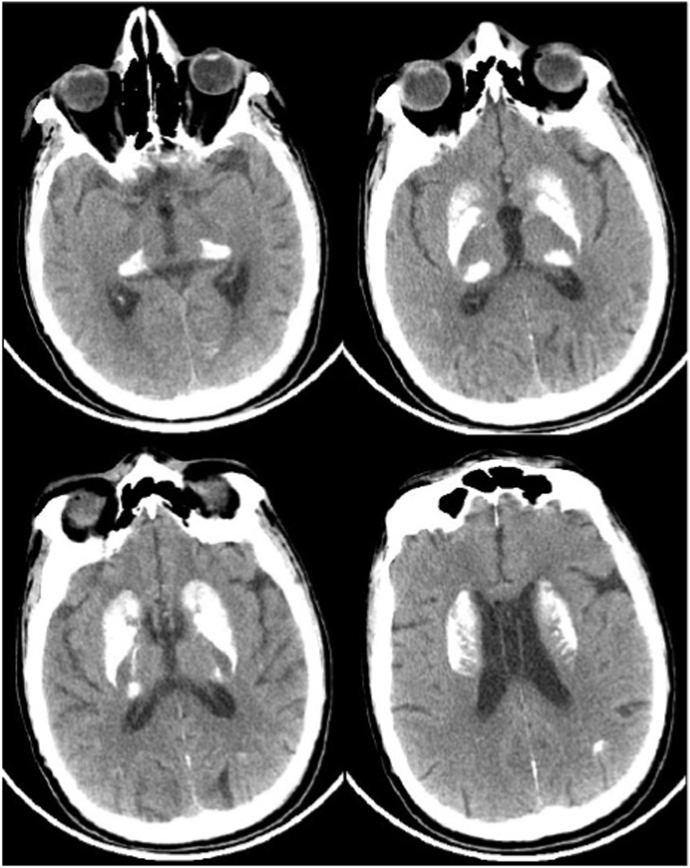
**Brain CT;** showed extensive symmetric bilateral basal ganglia calcifications.

**Fig. 2:**
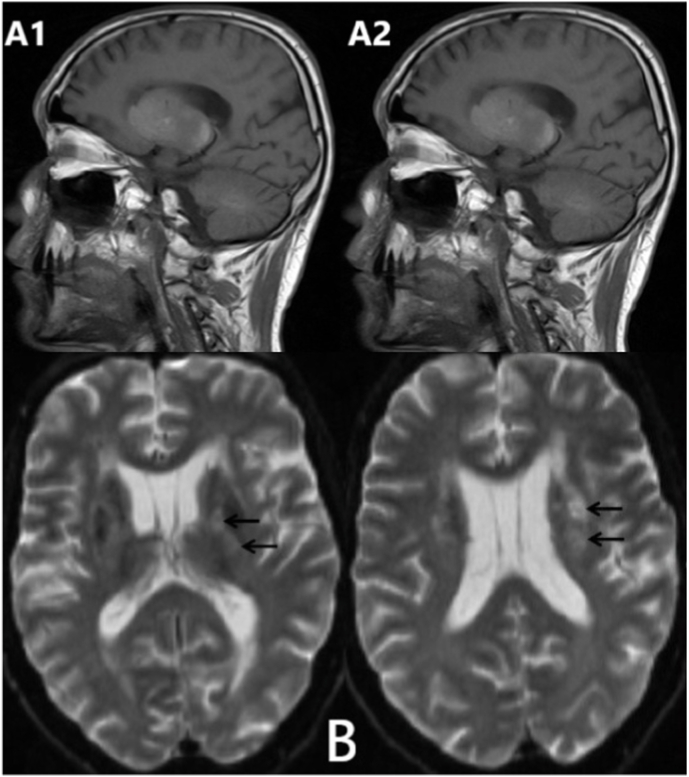
**Brain MRI; (A);** High-signal intensity lesions on T1-weighted consist with symmetric calcifications of the right (A1) and left (A2) basal ganglia.**(B);** High-signal intensity lesions on diffusion-weighted imaging consist with lacunar infarcts in the left caudate nucleus and corona radiate (black arrows).

**Fig. 3:**
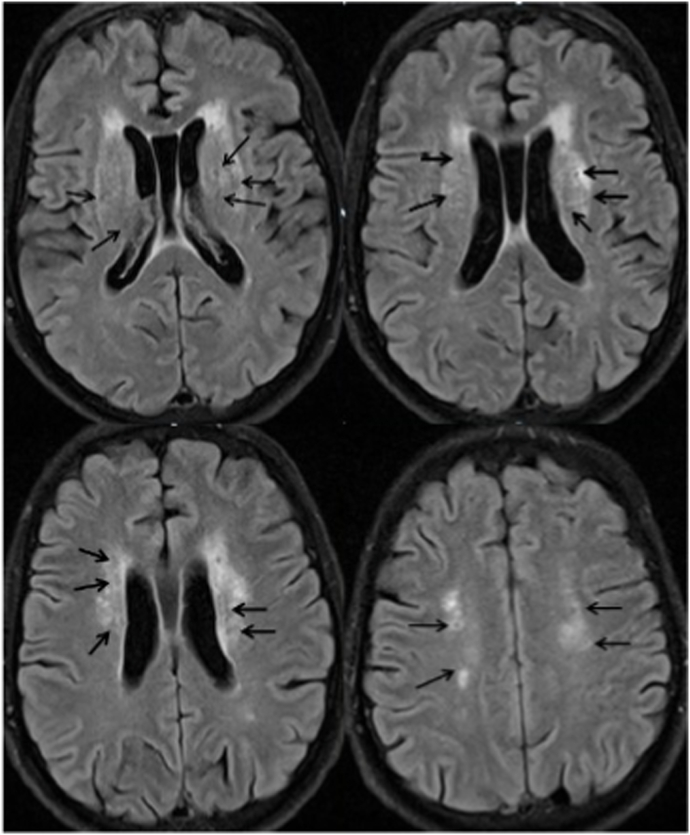
**Flair-weighted MRI;** White matter hyperintensities around the ventricles with central hypointense in some lesions correspond with periventricular leukomalacia.

First, we confirmed all previous elements of manifestations. PR was confirmed by previous records and contacting with the ophthalmologist, and bilateral SNHL by a previous audiogram. Also, a novel ophthalmologic examination is compatible with uveitis sequelae, which showed 1/10 visual acuity, posterior synechia, posterior cataract, cells in the anterior chambers, and mild ptosis. Migraine was confirmed by the history of unilateral pulsatile headache attacks with nausea, vomiting, and light aggravation. Brain MRI and CT showed symmetric basal ganglia calcifications with lacunar infarcts in the left caudate nucleus and corona radiate, and ischemic lesions in the white matter. Laboratory tests in the previous admissions show in Table 1 and neuroimaging in Fig. 1, Fig. 2, Fig. 3.

**Table 1:** Previous laboratory tests.

B 9	7	C3	124
B 12	256	C4	44
Ferritin	172	B2Micro	2
T- Sat	35%	Anti CMV (IgG)	Pos
Iron	75	Anti CMV (IgM)	Neg
TSH	2.5	P-ANCA	Neg
PTH	48	C-ANCA	Neg
Amylase	5	Anti-ds-DNA	Neg
Lipase	18	ASLO	Neg
Anti TPO	Neg	TST	Neg
Anti-thyroglobulin	Neg	Ur	20
HBS Ag	Neg	Cr	0.8
Anti HCV	Neg	ESR	15

T- Sat; Transferrin saturation, PTH; parathyroid hormone, TSH; thyroid stimulating hormone, Anti TPO; Anti-thyroid peroxidase antibodies, HBS Ag; Hepatitis B surface antigen, Anti HCV; *hepatitis C* antibody, C3–C4; complements, B2Micro; beta-2 microglobulin, CMV; *Cytomegalovirus,* P-ANCA; perinuclear anti-neutrophil cytoplasmic antibodies, C-ANCA; antineutrophil cytoplasmic antibodies, Anti-ds-DNA; anti-double stranded DNA, ASLO; Antistreptolysin O *test*, TST; Tuberculin skin test, Ur; urea, Cr; creatinine, ESR; erythrocyte sedimentation rate.

The broad multisystemic manifestations and family history point to inherited disorder rather than vasculitis or immune disorder. Only inherited disorders, that aligned with this entity and comprised all manifestations, are mitochondrial disorders, especially MELAS syndrome.

Where the genetic test for MELAS, mitochondrial DNA (mtDNA) mutation at A3242G point, is not available, additional tests (Table 2) are applied to exclude all other disorders and subsequently confirm the diagnosis. Systemic lupus erythematosus, antiphospholipid syndrome, Anti-neutrophil cytoplasmic autoantibody (*ANCA*)-associated *vasculitis*, viral infections, and other autoimmune disorders were all excluded (Table 1, Table 2). A peripheral blood smear was negative for neuroacanthocytosis, pathergy test was negative for Behcet disease and serum protein electrophoresis was negative for monoclonal gammopathy. Also, to investigate the edema, kidney tests revealed a nephrotic syndrome by 24-h urine test and urinalysis shows dysmorphic erythrocytes (Table 2), suggesting a glomerulopathy.

**Table 2:** Additional laboratory tests.

WBC	4	LA	Neg
HB	12	ACTH	28 N
PLT	195	Cortisol 8 AM	10.3 N
Alb	2.9	CSF-lactate	1.7 N
TP	5.5	CSF- Protein	86 H
Ur	21	Serum-lactate	1.9 N
Cr	0.9	Urine	
ALT	10	Protein	+
AST	18	HB	++
ESR	30	WBC	80
Ca	8.4	RBC	140
P	3	Crenated RBC	60 cells
CPK	46 N	Dysmorphic RBC	80 cells
LDH	287 N	Vol/24h urine	1500 ml
ANA	Neg	Prot/24h urine	3240 mg
Cardiolipin Ab (IgM-IgG)	Neg	Alb/24h urine	1254 mg
B2- GP1 (IgM-IgG)	Neg	Cr/24h urine	682 mg

WBC, white blood count; HB, hemoglobin; PLT, platelet; TP, total protein; ALB, albumin; AST, aspartate transaminase; ALT, alanine aminotransferase; Ca, calcium; P, phosphorus; CPK; Creatine Phosphokinase, LDH; lactate dehydrogenase; ANA; antinuclear antibodies, Ab; antibodies, B2- GP1; beta-2 glycoprotein 1 antibodies, LA; Lupus anticoagulant, ACTH; adrenocorticotropic hormone, CSF; *cerebrospinal fluid*, RBC; red blood cell.

**Fig. 4:**
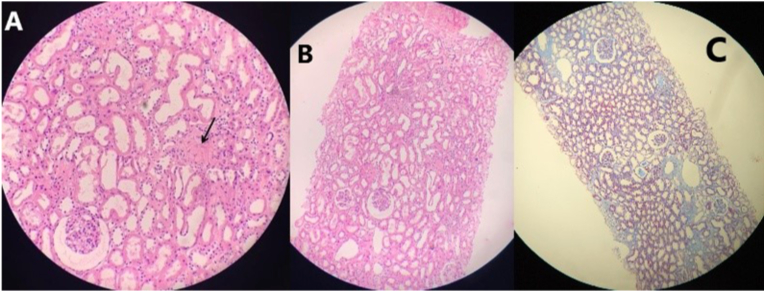
**Kidney biopsy;** PAS stain (A + B) and masson stain (C); showed a normal morphology with preserved glomerular architecture except a single hyalinized glomerulus (black arrow).

Kidney biopsy (Fig. 4) comprised of two fragments of renal cortex and medulla, containing 32 glomeruli and one is totally hyalinized. Glomerular architecture is preserved with normal thickness of glomerular basement membranes by PAS stain. The interstitium shows scattered inflammatory cells with normal masson stain. The tubular epithelium and blood vessels were normal. The immunofluorescence staining was not available. Muscle biopsy showed scant degenerated fibers with features of ragged red fibers by hematoxylin and eosin.

The patient was diagnosed with nephropathy in the context of MELAS syndrome and discharged on angiotensin-converting enzyme inhibitor (ACEI), furosemide, Co-enzyme Q10, and vitamin B2 (Riboflavin) [3]. The patient lost to follow up after being discharged. After a year, the patient referred to the nephrology clinic for edema exacerbation and did not committed to a previous prescription. A novel evaluation showed deterioration of kidney function, where serum Cr reached to1.7 mg/dl and was put on the same prescription with recommendations for serial follow up.

## 3 Discussion

The diagnosis of mitochondrial disorders is often challenging because of multisystem manifestations, dual genomic origins (nuclear and mitochondrial), and broad phenotypic heterogeneity [1].

Muscle biopsy remains an important tool for diagnosing a mitochondrial disease and is suggested when genetic testing cannot confirm the diagnosis and can be diagnostic even in cases where there is no evidence of myopathy from the history, physical examination, serum CK level, or electromyography. Muscle biopsy is most useful to confirm the diagnosis of mitochondrial myopathies when they are caused by a primary mitochondrial DNA defect since ragged red fibers are likely to be seen on the muscle histology. The main limitation in the diagnosis is that it may be normal or show only minimal abnormalities for certain mitochondrial defects [1]. However, myopathy is a cardinal manifestation of MELAS syndrome and a large proportion of patients probably have significant muscle involvement, this usually does not lead to significant functional impairment and may never be noticed or diagnosed [1,2]. Only one-half of patients have clinical evidence of myopathy and some patients have been misdiagnosed as polymyositis [1,3]. In the case of a 69‐year- old woman, who was first diagnosed with fibromyalgia; however this was followed by developing severe myalgia with a rapidly deteriorating mental status. The brain MRI demonstrated increased signals of multifocal lesions and the patient was misdiagnosed with CNS vasculitis and received corticosteroids. Thereafter, her serum creatine kinase levels began to rise and muscle biopsy revealed ragged red fibers, which led to the diagnosis of mitochondrial myopathy [7].

Here, the patient had no apparent muscle impairment or elevation of muscle enzymes, and the muscle histology showed minimal ragged red fibers. However, genetic detection was not available and the muscle biopsy, which was the favored method to make the diagnosis, only showed minimal abnormalities, this did not cause a barrier to diagnosis in the presence of other manifestations and family history.

While only some patients with MELAS syndrome present with mild pigmentary retinopathy (PR), the uvea is only rarely affected, and SNHL is typically mild and insidiously progressive [2,8]. Our patient was firstly presented with rare ophthalmologic manifestations, PR and uveitis. In addition to a latent SNHL and findings in brain imaging is similar to vasculitis. All these components might lead to misdiagnosing the status as vasculitis with frequent corticosteroids exposure. A few years later, the subsequently presenting of a nephrotic syndrome and precise family history suggest the mitochondrial disorder rather than vasculitis.

Parkinsonism is the most frequent hypokinetic disorder in patients with mitochondrial disorders. The majority of reports describing a Parkinson-like phenotype are associated with a mutation in nDNA genes but more rarely extrapyramidal features have been described in a few cases of mtDNA-related syndromes, such as MELAS [9]. Lactic acidosis is an important feature that is present in 94% of affected individuals [2]. CSF has lactate elevation in the majority of individuals and protein may be mildly elevated [2,3]. Here, the patient demonstrated typical signs and symptoms of Parkinson's disease with extrapyramidal features. CSF protein was elevated but the lactate was normal in serum and CSF, despite an obvious CNS involvement, which showed as extensive bilateral calcifications with lacunar infarcts of the basal ganglia and ischemic lesions in the white matter.

Numerous types of kidney involvement were reported in the context of MELAS syndrome. Although renal involvement in MELAS is rare, it may take the form of renal tubular acidosis, typically Fanconi syndrome; focal segmental glomerulosclerosis (FSGS), chronic kidney disease, and widespread interstitial fibrosis [3,[10], [11], [12], [13]]. Kidney biopsies to make FSGS diagnosis should contain a minimum of eight glomeruli and a few glomeruli (ie, fewer than 15) cannot confidently exclude the diagnosis of FSGS [14]. Histological finding of even a single glomerulus with segmental sclerosis or hyalinosis is enough to warrant a diagnosis of FSGS [15,16].

In our patient, however, the kidney biopsy comprised 32 glomeruli and was sufficient to make the histologic diagnosis; only showed a single hyalinized glomerulus despite the presence of the nephrotic syndrome. Since the known kidney pathology in MELAS was FSGS and the deterioration of kidney function in our patient, the later histologic findings were more likely pointed to FSGS than due to the biopsy sampling from an inappropriate site.

It is plausible that delays in diagnosis may be multi-faceted and heavily influenced by the onset of the disease which can occur at any age and clinicians may not be familiar with such a rare condition. Also, the broad phenotypic heterogeneity and the complexity of the disease may reduce the likelihood of a prompt diagnosis.

## 4 Conclusion

Here, we report a challenging case of MELAS syndrome affecting multiple organs with rare manifestations including uveitis, pigmentary retinopathy, parkinsonism, and nephropathy in the absence of lactic acidosis with unapparent muscle or hearing impairments. Since, clinicians might misdiagnose MELAS as vasculitis or other disorders, relevant and proper investigations should guide the diagnosis of these conditions to reduce the delay of diagnosis and ineffective treatments.

## Ethical approval

Written informed consent was obtained from the patient for publication of this case report and accompanying images, in line with local ethical approval requirements and in accordance with the helsinki declaration.

## Sources of funding

This research did not receive any specific Grant from funding agencies in the public, commercial, or not-for-profit sectors.

## Author contributions

Dr. Mohammad Alsultan wrote the manuscript, searched the literature, treat and follow up the patient and submitted the article.

Dr. Deema Alshaar: histopathologic diagnosis and write the histopathology sections of the article.

Badie Alkhouli: made study corrections and follow up the patient.

Qussai Hassan: made study corrections and supervisor of the research.

## Registration of research studies

1. Name of the registry: N\A.

2. Unique Identifying number or registration ID: N\A.

3. Hyperlink to your specific registration (must be publicly accessible and will be checked): N\A.

## Guarantor

The corresponding author is the guarantor of this manuscript.

## Provenance and peer review

Not commissioned, externally peer-reviewed.

## Consent

Written informed consent was obtained from the patient for publication of this case report and accompanying images. A copy of the written consent is available for review by the Editor-in-Chief of this journal on request.

## Declaration of competing interest

The author declares that they have no conflicts of interest regarding this study.

The author declares that it has not been published elsewhere and that it has not been submitted simultaneously for publication elsewhere.
